# The effects of melatonin on oxidative stress, inflammation, apoptosis and Nrf2/HO-1 in acrylamide-induced lung injury in rats

**DOI:** 10.1007/s00210-025-04292-8

**Published:** 2025-05-22

**Authors:** Aslıhan Atasever, Samet Tekin, İsmail Bolat, Merve Bolat, Yusuf Dağ, Burak ÇınaR, Emin Şengül, Serkan Yıldırım, Mohamad Warda, Fikret Çelebi

**Affiliations:** 1 Department of Veterinary Medicine, Çayırlı Vocational High School, Erzincan University, Erzincan, Turkey; 2 Department of Physiology, Faculty of Veterinary Medicine, Atatürk University, Erzurum, Turkey; 3 Department of Pathology, Faculty of Veterinary Medicine, Atatürk University, Erzurum, Turkey; 4 Department of Pharmacology, Atatürk University Faculty of Medicine, Erzurum, Turkey; 5 Department of Biochemistry, Faculty of Veterinary Medicine, Cairo University, Giza, Egypt; 6 Department of Pathology, Faculty of Veterinary Medicine, Kyrgyzs-Turkish Manas University, Bishkek, Kyrgyzstan

**Keywords:** Lung, Acrylamide, In silico, HO-1, Nrf-2

## Abstract

This study is to investigate the effects of melatonin on lung inflammation, oxidative stress, apoptosis, tissue damage, and MT1 and MT2 receptors in acrylamide-induced lung toxicity. Fifty male rats were randomly divided into five groups. The control group received distilled water orally for 11 days, while the acrylamide group received acrylamide (50 mg/kg, i.g.) for 11 days. The MEL10 + ACR and MEL20 + ACR groups received intraperitoneal injections of melatonin at doses of 10 mg/kg and 20 mg/kg, respectively, followed by acrylamide (50 mg/kg, i.g.) administered 1 h after melatonin injection. The MEL20 group received melatonin injections (20 mg/kg) for 11 days. Lung tissues collected at the end of the study underwent biochemical, histopathological, immunohistochemical, immunofluorescence, and in silico analyses. Acrylamide caused oxidative stress, inflammation, apoptosis, and tissue damage in the lungs. Melatonin treatment alleviated acrylamide-induced lung damage by exhibiting antioxidant, anti-inflammatory, and anti-apoptotic effects. Melatonin significantly improved the histopathological changes caused by acrylamide in lung tissue. Melatonin may have protective effects on health by regulating cellular processes such as oxidative stress, antioxidant enzyme activity, inflammation, and apoptosis through MT1 and MT2 receptors. Melatonin mitigates oxidative stress, inflammation, apoptosis, and tissue damage in acrylamide-induced lung injury in rats.

## Introduction

Acrylamide (ACR) is a synthetic toxic chemical compound produced in the 1950 s (Kacar et al. 2019), widely used in industrial fields (Tareke et al. 2002), and found in baked and fried foods such as potato chips and cookies (Dutta et al. 2015). In 2002, it was first discovered that ACR is also formed in high-temperature processed foods, suggesting that human exposure to ACR can occur through dietary intake. Consequently, the number of studies on ACR toxicity increased significantly during those years (Tareke et al. 2002). ACR can enter the body through food, air, and skin, and blood levels rise shortly after exposure. It forms adducts with hemoglobin and can be transported to different organs. Its biotransformation occurs in hepatocytes and other tissues with high cytochrome P450 activity. ACR is metabolized via cytochrome P450 (CYP2E1) and conjugation with glutathione (GSH). Cytochrome P450 converts ACR into glycidamide (GA). Both ACR and GA conjugate with GSH to form GSH adducts. These are precursors of mercapturic acid metabolites and are excreted through urine. GA, which causes cellular damage, is genotoxic. Electron transfer between CYP2E1 and reductase can facilitate the formation of reactive oxygen species (ROS). Oxidative stress and DNA damage mechanisms play significant roles in ACR toxicity. Although toxicity primarily occurs in the liver, where ACR biotransformation is intense, organs with high cytochrome P450 activity are also sensitive to ACR's toxic effects (Batoryna et al. 2019).

It has been proven that acrylamide (ACR) has various toxic effects. ACR is a carcinogenic (Dearfield et al. 1995), neurotoxic (Gur et al. 2021), genotoxic (Yang et al. 2005), reprotoxic (Sakamoto & Hashimoto 1986), and mutagenic (Ishii et al. 2015; Manjanatha et al. 2015) agent, which explains its multi-organ toxicity. ACR also causes lung toxicity (Yesildag et al. 2022). The lungs play a role in the metabolism of inhaled toxins as they not only perform the biotransformation of inhaled foreign substances but also have high cytochrome P450 activity. Therefore, one of the toxic and mutagenic effects of ACR in the lungs may stem from the biotransformation process and the resulting oxidative imbalance (Batoryna et al. 2019). Inhalation of ACR can directly irritate and damage the respiratory system. Regardless of the exposure route, ACR exposure leads to oxidative stress and inflammation due to the depletion of antioxidants such as glutathione (GSH). This systemic effect can contribute to lung dysfunction and respiratory diseases. ACR has been reported to cause lung dysfunction and increased systemic inflammation in the population (Wang et al. 2021). For this reason, various antioxidants have been investigated in animal models to mitigate the effects of ACR-induced lung injury.

Melatonin (MEL), a hormone produced by the pineal gland, has antioxidant, anti-apoptotic, anti-cancer, and anti-aging properties, and it also affects the immune system (Macit et al. 2013). There is substantial evidence that MEL and its metabolites exert protective effects on DNA, lipids, and proteins as a result of both endogenous and exogenous free radical production processes. Moreover, MEL not only neutralizes various free radicals and reactive oxygen and nitrogen species directly, but it also enhances antioxidant activity by activating antioxidant enzymes such as superoxide dismutase, glutathione peroxidase, and glutathione reductase. MEL’s powerful protective effect against oxidative stress is further strengthened by its ability to cross all biological membranes. This property enables MEL to concentrate in the cell nucleus, protecting DNA from free radical damage (Tozan-Beceren et al. 2012). Furthermore, MEL can significantly reduce the levels of pro-apoptotic factors and increase the levels of anti-apoptotic factors. MEL’s anti-inflammatory effect is mediated by regulating the expression levels of inflammatory cytokines, inflammatory mediators, and inflammatory cell infiltration (Sheikholeslami et al. 2021). MEL also shows a protective role against lipid peroxidation and cellular damage by inhibiting the production of malondialdehyde (MDA) (Ahmadiasl et al. 2014).

MEL has receptors known as MT1 and MT2. These receptors are G protein-coupled receptors. G proteins are involved in regulating the immune system and various cellular signal transduction processes (Wang & Gao 2021). MEL receptors are widely distributed in both the central nervous system and peripheral tissues. Activation of the different MEL receptors, named MT1 and MT2, leads to various physiological responses in the body, including regulation of the sleep–wake cycle, balancing the immune system, and controlling the cardiovascular system. In short, many of MEL’s physiological effects occur through the activation of MT1 and MT2 receptors (Zlotos et al. 2014). Studies conducted in light of this information have shown that MEL provides protection against ischemia/reperfusion (I/R) damage in various organs, including the brain, heart, kidneys, and liver (Zhou et al. 2015). Considering these aspects, we decided to examine the effects of ACR administered via oral gavage in rats on the lungs, associating them with antioxidant, anti-inflammatory, and anti-apoptotic functions through biochemical, histopathological, immunohistochemical, immunofluorescent, and in silico analyses.

## Materials and methods

### Chemicals used in the study

ACR (≥ 99%) was provided by Sigma Chemical Co. (St. Louis, MO), and melatonin (MEL) supplement (≥ 99%) was provided by Alfa Aesar. Sevoflurane (Sevorane %100 sıvı, Abbvie Laboratories, İstanbul, Turkey) was used to anesthetize rats for lung tissue removal at the end of the experimental study. Catalase (CAT), glutathione peroxidase (GPx), glutathione (GSH), heme oxygenase 1 (HO-1), interleukin-1 beta (IL-1β), interleukin-10 (IL-10), malondialdehyde (MDA), nuclear factor erythroid 2-related factor 2 (Nrf2), nuclear factor kappa (NF-κB), superoxide dismutase (SOD), tumor necrosis factor alpha (TNF-α), caspase 3 (CASP3), inducible nitric oxide synthase (iNOS; NOS2), interleukin-6 (IL-6), cyclooxygenase-2 (COX-2), p38 mitogen-activated protein kinases (p38-MAPK), and myeloperoxidase (MPO) enzyme-linked immunosorbent assay (ELISA) kits were purchased from BT-LAB.

### Animals

In the study, 50 adult male Sprague–Dawley rats (approximately 240–270 g) were obtained from the Experimental Research and Application Center of Atatürk University in Erzurum. The rats were housed at a temperature of 21 ± 2°C, with 55 ± 5% humidity, under a 12-h-light/dark cycle. They were provided with water and pellet feed ad libitum (Bayramoğlu Feed and Flour Industry Trade Inc., Erzurum, Turkey).

### Experimental design

At the beginning of the experiment, all rats were weighed, their weights were standardized, and they were randomly divided into five groups. After the groups were assigned, they were named according to the study protocol, and the protocol, which lasted for 11 days, was followed as specified.

1. CONTROL group: Rats in this group were administered 1 mL of distilled water intragastrically for 11 days.
2. ACR group: Rats in this group were administered ACR (50 mg/kg) intragastrically for 11 days (Ibaokurgil et al. 2023).
3. MEL10 + ACR group: This treatment group received 10 mg/kg MELintraperitoneally for 11 days. One hour after each MEL injection, ACR (50 mg/kg, i.g.) was administered.
4. MEL20 + ACR group: This treatment group received 20 mg/kg melatonin intraperitoneally for 11 days. One hour after each MEL injection, ACR (50 mg/kg, i.g.) was administered (Yeleswaram et al. 1997).
5. MEL20 group: Rats in this group were administered MEL (20 mg/kg, i.p.) for 11 days (Ibaokurgil et al. 2023).

At the end of the experimental procedures, the live body weights of the rats were measured. After all the rats were decapitated under light sevoflurane anesthesia, the lung tissues were removed, and their weights were recorded. After weighing the stomach tissues, the lungs of the rats from each group were washed with physiological serum for histopathological examinations, then immediately placed in formaldehyde (10%) and embedded in paraffin blocks and preserved. For biochemical analyses, the lungs from each group were washed with physiological saline and stored at − 80 °C until use.

### Lung tissue homogenization

Equal amounts (80 mg) of lung tissue samples were placed in screw-cap tubes, and 1500 µL of phosphate-buffered saline (PBS) solution was added. The tissue homogenization was then performed using a Magna Lyser homogenizer. After homogenization was performed at 5000 rpm for approximately 10 min, the samples were centrifuged at 5000 rpm for 10 min. The supernatants were carefully transferred to clean Eppendorf tubes for further analysis.

### Analysis of oxidative stress markers

Lung tissue supernatants were obtained for the analysis of biochemical markers. The levels of MDA (Cat.No E0156Ra), GSH (Cat.No E0259Ra), Nrf2 (Cat.No E1083Ra), and HO-1 (Cat.No E0676Ra) in the lung tissue supernatants, as well as the activities of SOD (Cat.No E0168Ra), GPx (Cat.No), and CAT (Cat.No E0869Ra), were measured using ELISA kits according to the manufacturer’s instructions. The analysis was performed using an ELISA plate reader (Bio-Tek, Winooski, VT, USA), and absorbance was read at a wavelength of 450 nm.

### Analysis of inflammation markers

The levels of TNF-α (Cat.No E0764Ra), IL-1β (Cat.No E0119Ra), NF-κB (Cat.No E0287Ra), IL-10 (Cat.No E0108Ra), iNOS (Cat.No E0740Ra), IL-6 (Cat.No E0135Ra), COX-2 (Cat.No E0296Ra), MPO (Cat.No E0574Ra), and p38 MAPK (Cat.No E2473Ra) in the lung tissue supernatants were measured using ELISA kits according to the manufacturer’s instructions. The analysis was performed using an ELISA plate reader (Bio-Tek, Winooski, VT, USA), and absorbance was read at a wavelength of 450 nm.

### Analysis of apoptosis markers

The levels of CASP3 (Cat.No E1648Ra) in the lung tissue supernatants were measured using ELISA kits according to the manufacturer’s instructions. The analysis was performed using an ELISA plate reader (Bio-Tek, Winooski, VT, USA), and absorbance was read at a wavelength of 450 nm.

### Histopathological examination

At the end of the evaluation, the collected lung tissue samples were fixed in a 10% formaldehyde solution for 48 h and embedded in paraffin blocks following routine tissue processing procedures. Sections of 4 µm thick were taken from each block and stained with hematoxylin–eosin (HE) for histopathological examination under a light microscope (Olympus BX 51, JAPAN). The sections were assessed based on their histopathological characteristics as absent (−), mild (+), moderate (+ +), and severe (+ + +) (Karabulut Uzunçakmak et al. 2023).

### Immunohistochemical examination

For immunoperoxidase staining, tissue sections placed on adhesive (poly-L-lysine) slides were deparaffinized and dehydrated. They were then treated with 3% H_2_O_2_ for 10 min to inactivate endogenous peroxidase. The tissues were subsequently boiled in a 1% antigen retrieval solution (citrate buffer, pH 6.1, 100 ×) and left to cool at room temperature. To prevent nonspecific background staining, the sections were incubated with a protein block for 5 min. After that, the tissues were incubated with the primary antibody (Bax Cat No: sc-7480, dilution 1/100, US) according to the manufacturer’s instructions.The 3,3′-diaminobenzidine (DAB) chromogen was used as the chromogen for the tissues. The stained sections were examined under a light microscope (Zeiss AXIO, GERMANY) (Bolat et al. 2024).

### Double immunofluorescence examination

For immunoperoxidase staining, tissue sections placed on adhesive (poly-L-lysine) slides were deparaffinized and dehydrated. They were then treated with 3% H_2_O_2_ for 10 min to inactivate endogenous peroxidase. The tissues were subsequently boiled in a 1% antigen retrieval solution (citrate buffer, pH 6.1, 100 ×) and left to cool at room temperature. To prevent nonspecific background staining, the sections were incubated with a protein block for 5 min. The tissues were then incubated with the primary antibody (JNK Cat No: sc-514539, dilution 1/100, US) according to the manufacturer’s instructions. The secondary marker used was an immunofluorescent secondary antibody (FITC Cat No: ab6785, dilution 1/1000), and the tissues were kept in the dark for 45 min. Afterward, the tissues were incubated with a second primary antibody (Caspase 3 Cat No: sc-56053, dilution 1/100, US) according to the manufacturer’s instructions. The secondary marker used was an immunofluorescent secondary antibody (Texas Red Cat No: ab6719, dilution 1/1000 UK), and the tissues were kept in the dark for 45 min. Then, DAPI (Cat No: D1306, dilution 1/200 UK) with a mounting medium was applied to the sections, and they were left in the dark for 5 min. Afterward, the sections were covered with coverslips. The stained sections were examined under a fluorescence attachment microscope (Zeiss AXIO, GERMANY) (Sulukan et al. 2023).

### In silico analysis of melatonin on MT1 and MT2 receptors

In this study, in silico molecular docking and molecular dynamics analyses of MEL on MT1 and MT2 receptors will be discussed. All analyses were performed using the licensed Schrödinger Maestro 2023/4 version.

#### Molecular docking analysis

Molecular docking analysis was conducted to identify the potential interactions of MEL with MT1 and MT2 receptors. Molecular docking was performed with a rigid protein and flexible ligand. Ligand-receptor interaction energies and binding positions were obtained using Glide (Schrödinger Suite) (Fig. 9) (Hasanat et al. 2017).

#### Surface and 2D/3D visualization

The 2D and 3D visualization tools of Schrödinger Maestro were used for the visualization of the analyses. Molecular structures, surface properties, and interaction regions were analyzed in detail (Fig. 9) (Chaudhari & Bari 2016).

#### Pharmacophore mapping analysis

Pharmacophore mapping analysis was used to determine the pharmacophore features of MEL on MT1 and MT2 receptors. This analysis was performed to define the receptor-specific interaction properties of the ligand (Fig. 9) (Kumar et al. 2019).

#### Molecular dynamics analysis

Molecular dynamics analysis was conducted to evaluate the dynamic behavior of interactions between MEL and MT1 and MT2 receptors. Using Desmond Molecular Dynamics Suite (Schrödinger), the behavior of ligand-receptor complexes over time was analyzed (Cai et al. 2021).

These analytical processes were designed to ensure a comprehensive evaluation of in silico studies. The obtained results will contribute to understanding the interaction mechanisms of MEL with MT1 and MT2 receptors and provide insights for potential therapeutic applications.

### Statistical analysis

The quantitative data obtained at the end of the study were analyzed using one-way ANOVA followed by the Tukey test. These analyses were performed using the GraphPad Prism 8.0.1 statistical software. A *p*-value of < 0.05 was considered significant.

For histopathological examinations, the statistical analysis was performed using the SPSS 13.0 software, and a *p*-value of < 0.05 was considered significant.The Duncan’s test was used for inter-group comparisons. The non-parametric Kruskal–Wallis test was used to detect group interactions, and the Mann–Whitney *U* test was used to determine differences between groups.

To determine the intensity of positive staining in the images obtained from immunohistochemical and immunofluorescent staining, five random areas were selected from each image, and evaluations were performed usingthe ZEISS Zen Imaging Software. The data were statistically defined as the mean and standard deviation (mean ± SD) for area percentage. One-way ANOVA followed by Tukey’s test was performed to compare the immunoreactive cells and immunopositive stained areas with healthy controls (GraphPad Prism).

### Ethical statement

The protocol for this study was approved by the Local Ethics Committee for Animal Experiments of Atatürk University (HADYEK: 2023/05).

## Results

### Effects of ACR and MEL treatments on body and lung weights

In this study, where we investigated the potential protective effects of MEL in the ACR-induced lung toxicity model, body and lung weights were evaluated among the groups. No differences were observed in the weights of rats among the groups before the experiment (*p* > 0.05). At the end of the experimental study, it was found that the average body weights of rats in the ACR and MEL10 + ACR groups were significantly reduced compared to the control group (*p* < 0.05). No significant difference in the average body weights of rats was found between the MEL20 + ACR and MEL20 groups and the control group (*p* > 0.05). The average lung weights of rats did not show any significant differences among the experimental groups (*p* > 0.05) (Table 1).

**Table 1:** Body and lung weights of rats in the experimental groups (*n* = 10)

Parameters	Control	ACR	MEL10 + ACR	MEL20 + ACR	MEL20
Initial body weight (g)	241.53 ± 25.56^a^	235.41 ± 16.32^a^	232.86 ± 16.18^a^	226.86 ± 14.17^a^	223.29 ± 12.03^a^
Final body weight (g)	264.57 ± 15.24^a^	206.57 ± 24.96^b^	218.86 ± 12.32^b^	232.86 ± 18.32^ab^	236.14 ± 13.71^ab^
Lung weight (mg)	1.70 ± 0.0.14^a^	1.24 ± 0.11^b^	1.51 ± 0.03^ac^	1.53 ± 0.14^ac^	1.57 ± 0.10^ac^

Statistical differences are present between values expressed with different letters within the same row.

^a–b^*p* < 0.05; *n* = 10; ± SEM)

### Effects of melatonin on lipid peroxidation and antioxidant enzymes

**Fig. 1:**
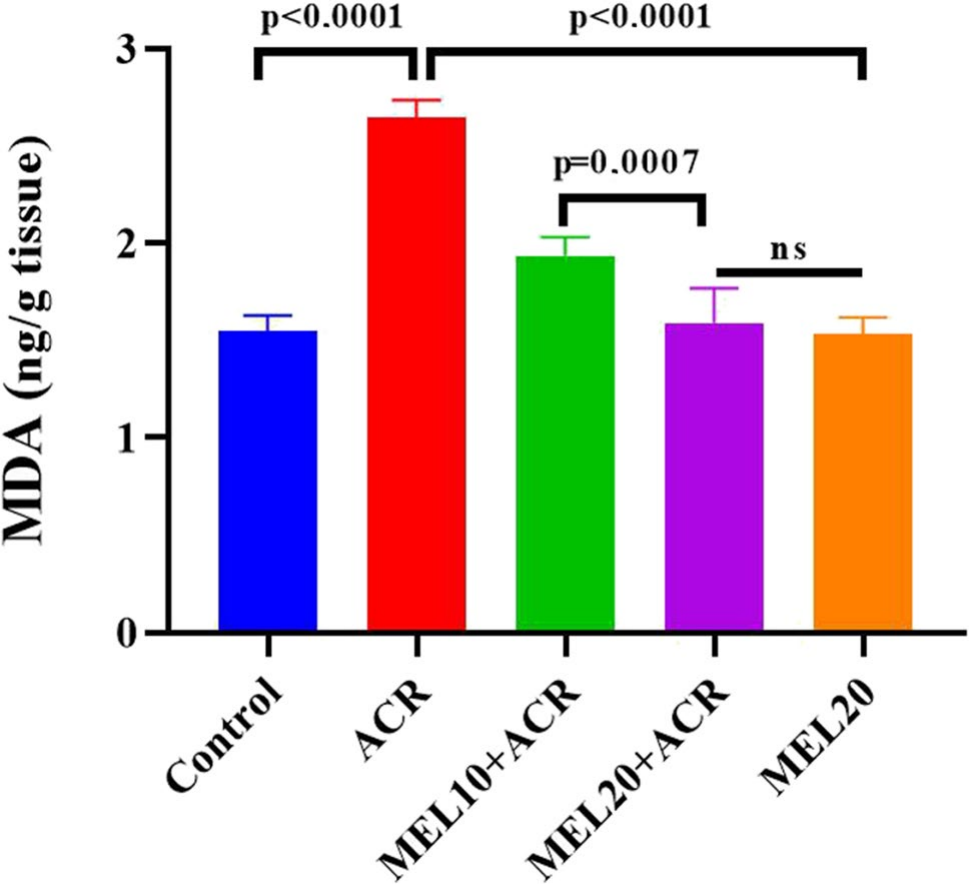
Lung MDA levels in experimental groups (*n* = 10). Results are expressed as mean ± SEM

As shown in Fig. 1, the MDA level in lung tissue was significantly increased in the ACR group compared to the control, MEL20 + ACR, and MEL20 groups. In the MEL10 + ACR group, the MDA level decreased compared to ACR, but this reduction was not as effective as in the MEL20 + ACR group. When comparing the control group with MEL10 + ACR, the MDA level was found to be statistically significantly higher in the MEL10 + ACR group (*p* = 0.0002). No significant difference in MDA levels was observed between the MEL20 + ACR, MEL20, and control groups (*p* > 0.05).

**Fig. 2:**
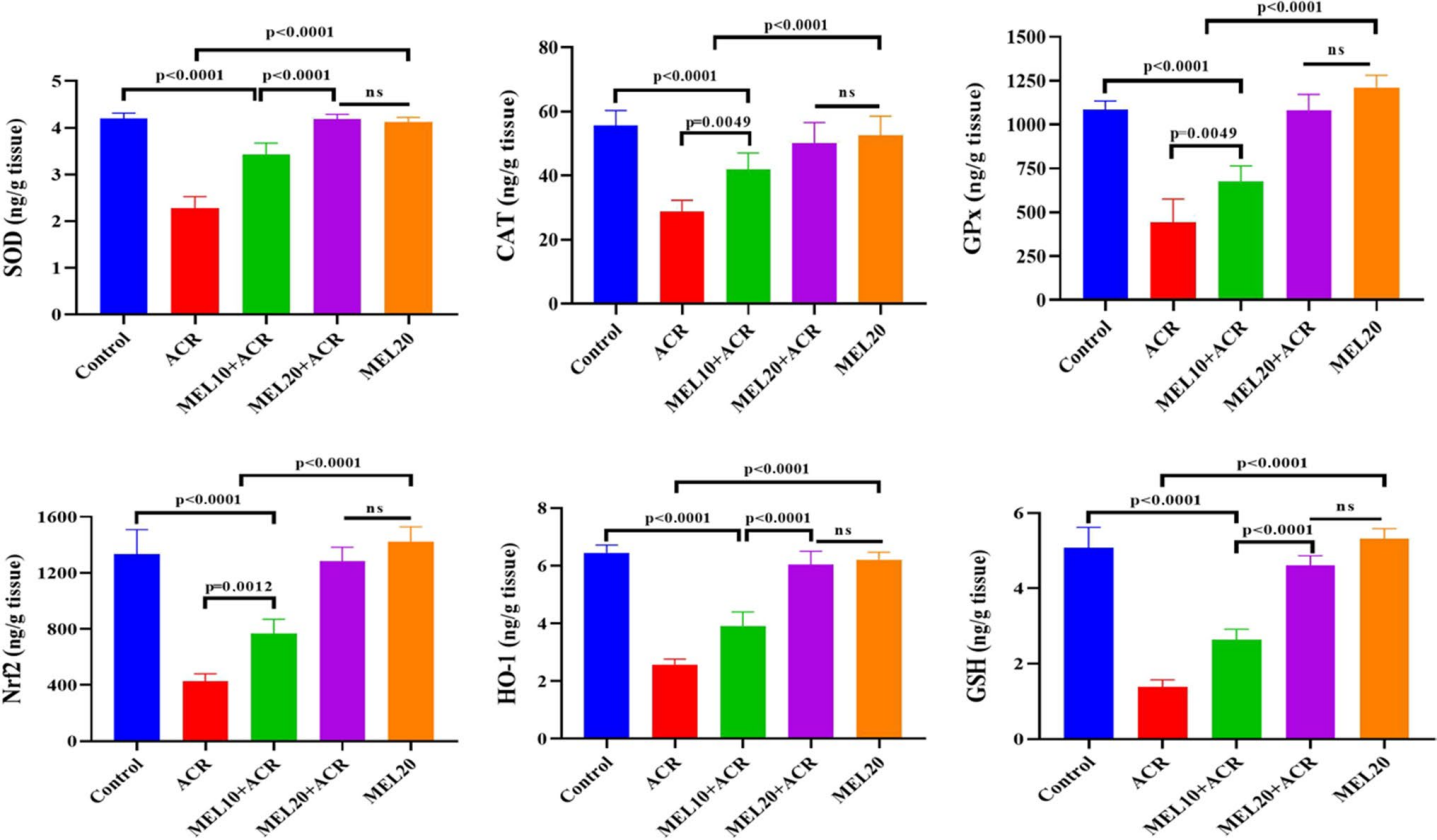
Lung SOD, CAT, GPx, Nrf2, HO-1 activities, and GSH levels in experimental groups (*n* = 10). Results are expressed as mean ± SEM

As shown in Fig. 2, the activities of SOD, CAT, GPx, Nrf2, HO-1, and the level of GSH were significantly decreased in the ACR toxicity group compared to the control, MEL20 + ACR, and MEL20 groups. In the MEL10 + ACR group, the activities of SOD, CAT, GPx, Nrf2, HO-1, and GSH levels were increased compared to the ACR group; although, this increase was not as effective as in the MEL20 + ACR group. When comparing the control group to the MEL10 + ACR group, a statistically significant difference was observed. The activities of SOD, CAT, GPx, Nrf2, HO-1, and the level of GSH in the MEL20 + ACR and MEL20 groups were significantly higher than in the ACR group. However, there were no significant differences when comparing the control group with the MEL20 + ACR and MEL20 groups (*p* > 0.05).

### Effect of melatonin on lung ınflammation

**Fig. 3:**
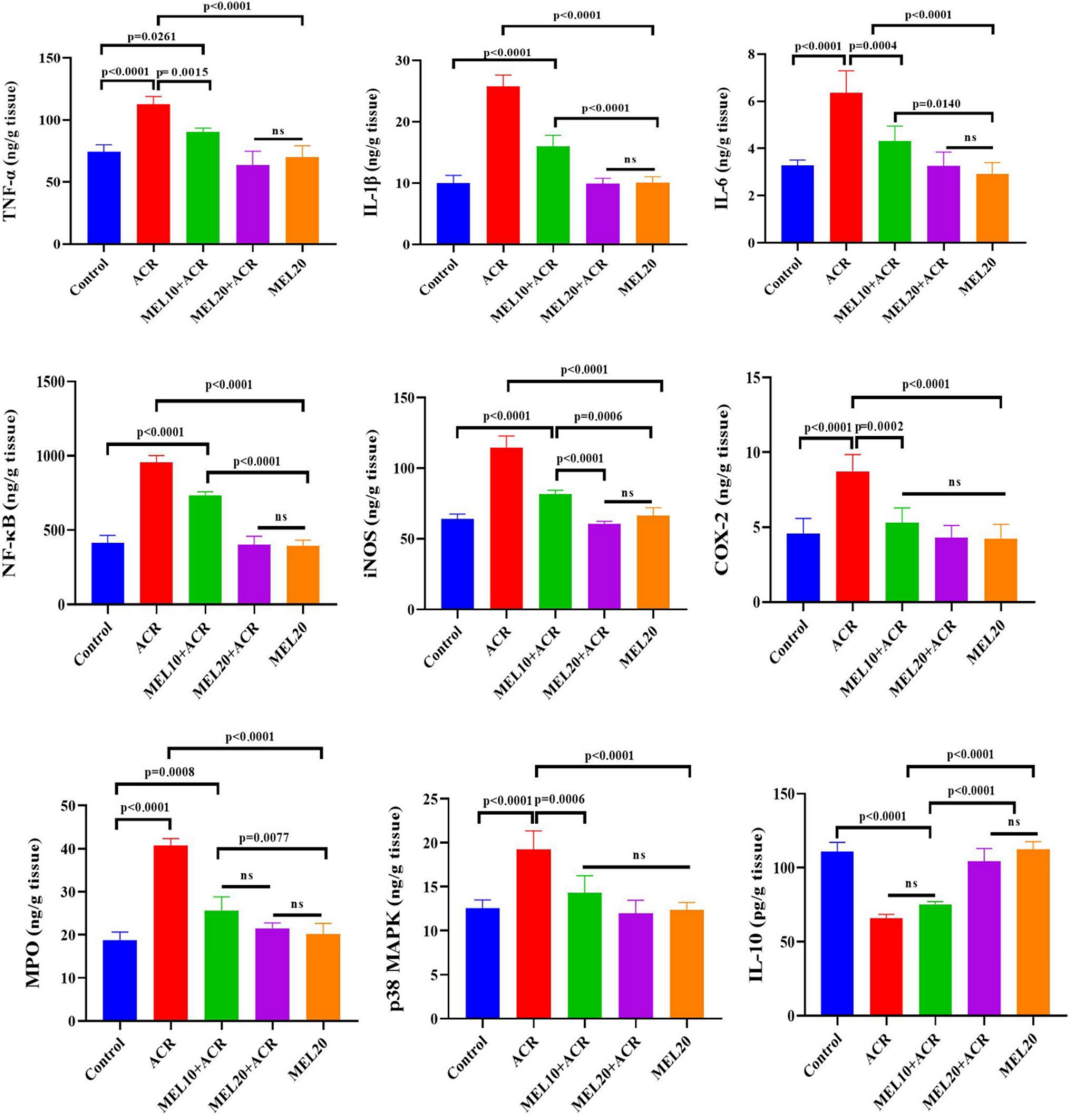
Lung TNF-α, IL-1β, IL-6, NF-κB, iNOS, COX-2, MPO, p38 MAPK, and IL-10 activities in experimental groups (*n* = 10). Results are expressed as mean ± SEM

As shown in Fig. 3, when examining the TNF-α, IL-1β, IL-6, NF-κB, iNOS, COX-2, MPO, and p38 MAPK activities, a significant increase was observed in the ACR group compared to the control, MEL20 + ACR, and MEL20 groups. In the MEL10 + ACR group, the levels of TNF-α, IL-1β, IL-6, NF-κB, iNOS, COX-2, MPO, and p38 MAPK were reduced compared to the ACR group, but this reduction was not as effective as in the MEL20 + ACR group. In the MEL20 + ACR and MEL20 groups, a significant reduction was observed in these inflammatory markers compared to the ACR group. No significant differences were found in TNF-α, IL-1β, IL-6, NF-κB, iNOS, COX-2, MPO, and p38 MAPK activities in the MEL20 + ACR and MEL20 groups compared to the control group (*p* > 0.05). IL-10 activity was significantly decreased in the ACR toxicity and MEL10 + ACR groups compared to the control, MEL20 + ACR, and MEL20 groups. IL-10 activity in the MEL20 + ACR and MEL20 groups was significantly increased compared to the ACR and MEL10 + ACR groups. No significant differences were found when comparing the control group with the MEL20 + ACR and MEL20 groups (*p* > 0.05).

### Effects of melatonin on apoptosis enzymes

**Fig. 4:**
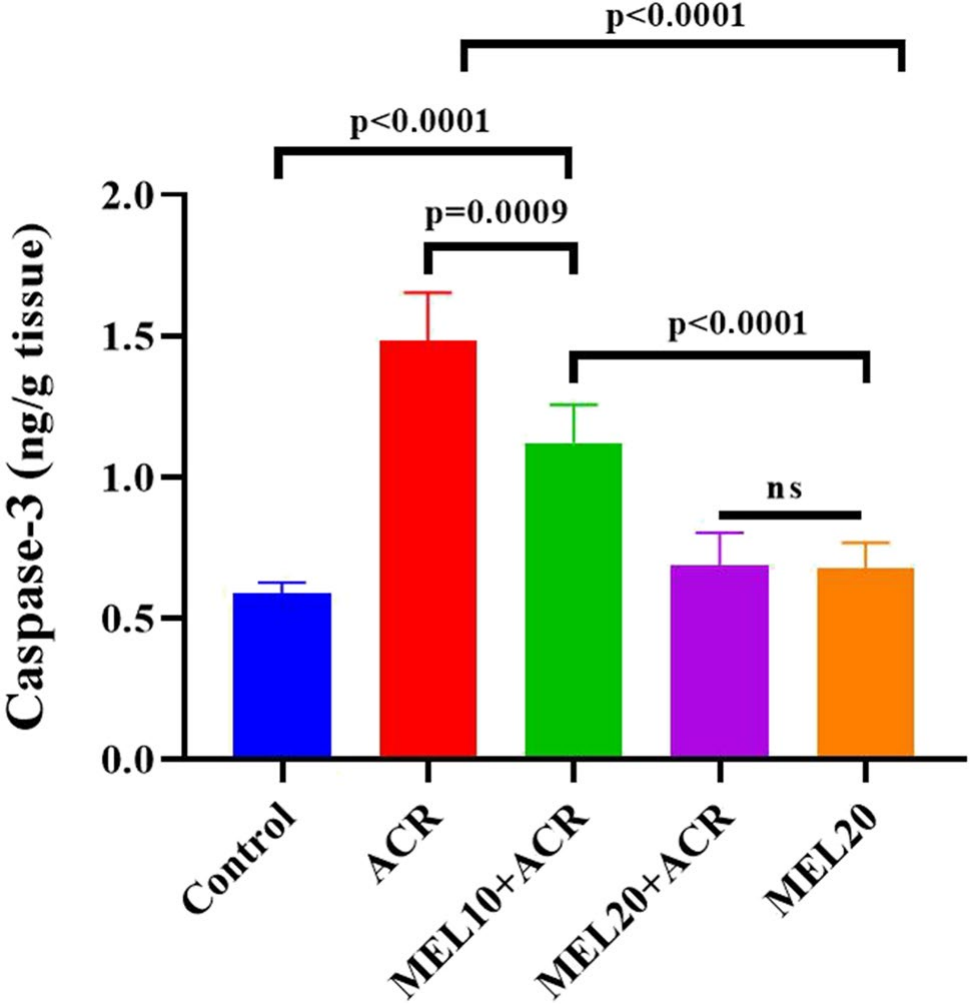
Lung CASP3 activity in experimental groups (*n* = 10). Results are expressed as mean ± SEM

As seen in Fig. 4, lung tissue CASP3 activity was significantly increased in the ACR group compared to the control, MEL20 + ACR, and MEL20 groups. In the MEL10 + ACR group, CASP3 activity decreased compared to ACR, but this decrease was not as effective as in the MEL20 + ACR group. When comparing the control and MEL10 + ACR groups, CASP3 activity was found to be significantly higher in the MEL10 + ACR group. CASP3 levels did not show significant differences compared to the control group in the MEL20 + ACR and MEL20 groups (*p* > 0.05).

### Histopathological findings

**Fig. 5:**
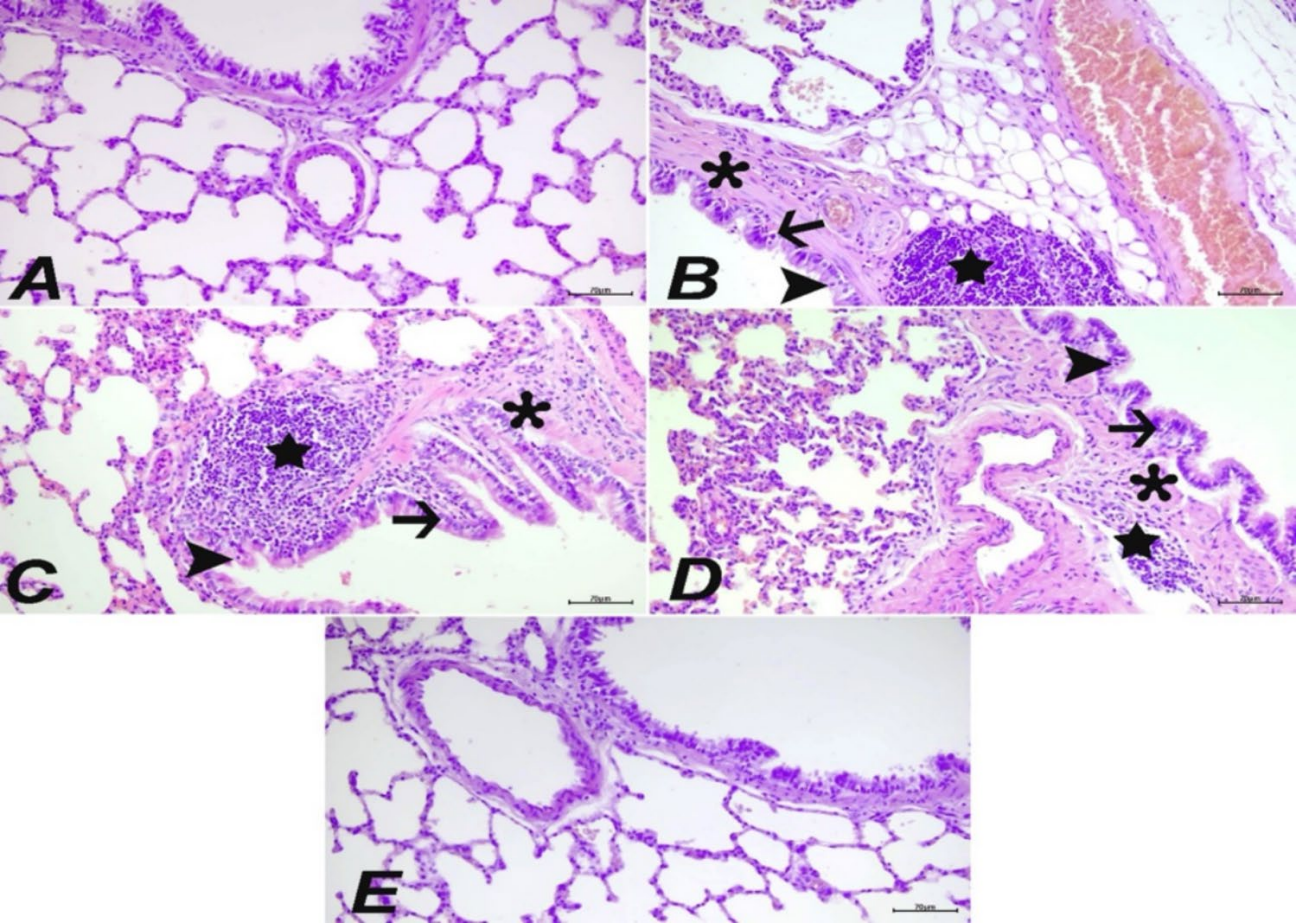
Lung tissue, control (**A**), ACR (**B**), MEL10 + ACR (**C**), MEL20 + ACR (**D**), and MEL20 (**E**). Degeneration (arrows) and necrosis (arrowheads) in bronchial epithelial cells, mononuclear cell infiltrations (stars), and fibromuscular hypertrophy (asterisks), H&E, bar: 70 µm

When lung tissues from the control and MEL20 groups were examined histopathologically, it was determined that the tissues had a normal histological structure (Fig. 5A and E). In the ACR group, histopathological examination of the lung tissues revealed severe degeneration and necrosis in the bronchial, bronchiolar, and alveolar epithelial cells. Severe mononuclear cell infiltration around the bronchi and bronchioles, as well as in the interalveolar areas, and severe thickening in the interalveolar septum were observed. Severe fibromuscular hypertrophy was detected around the bronchi and bronchioles (Fig. 5B). In the MEL10 + ACR group, severe degeneration and moderate necrosis were observed in the bronchial, bronchiolar, and alveolar epithelial cells. Severe mononuclear cell infiltration around the bronchi and bronchioles, as well as in the interalveolar regions, and severe thickening in the interalveolar septum were noted. Moderate fibromuscular hypertrophy was seen around the bronchi and bronchioles (Fig. 5C). In the MEL20 + ACR group, moderate degeneration and mild necrosis were observed in the bronchial, bronchiolar, and alveolar epithelial cells. Mild mononuclear cell infiltration around the bronchi and bronchioles, as well as in the interalveolar regions, and mild thickening in the interalveolar septum were noted. Mild fibromuscular hypertrophy was detected around the bronchi and bronchioles (Fig. 5D). Scoring of histopathological findings and statistical analysis results are presented in Fig. 8.

### Immunohistochemical and immunofluoresence findings

**Fig. 6:**
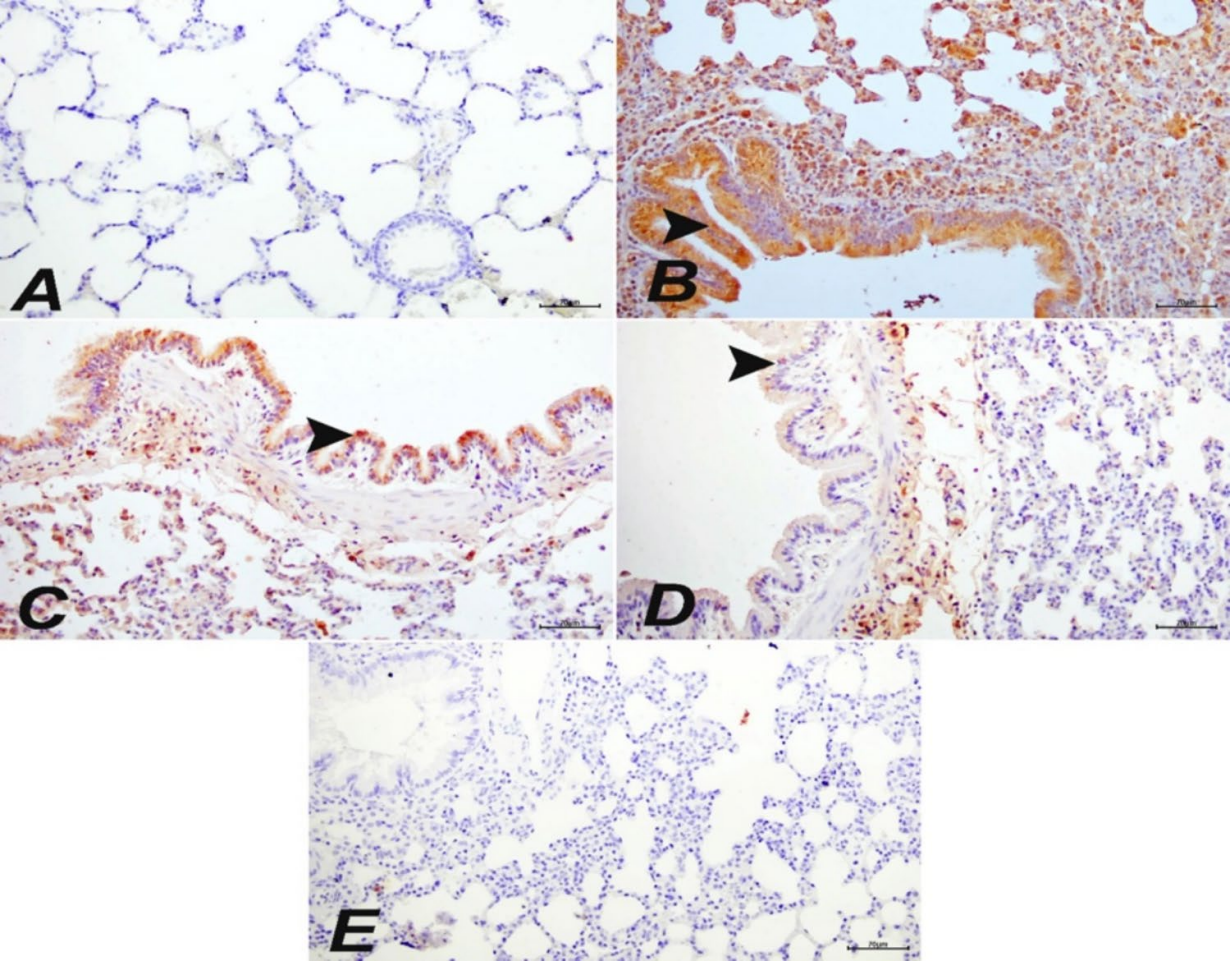
Lung tissue, control (**A**), ACR (**B**), MEL10 + ACR (**C**), MEL20 + ACR (**D**), and MEL20 (**E**). Intracytoplasmic BAX expressions in bronchial, bronchiolar, and alveolar epithelial cells (arrowheads), IHC-P, bar: 70 µm

**Fig. 7:**
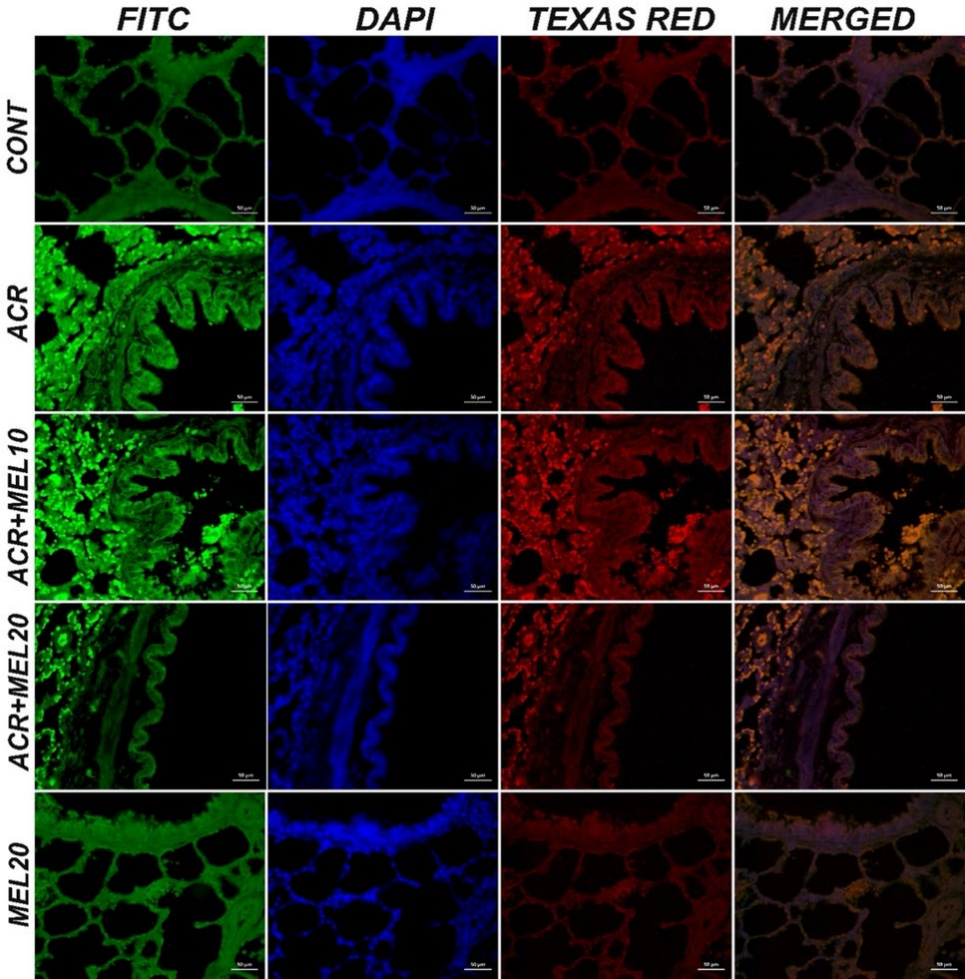
Lung tissue, JNK (FITC) and CASP3 expressions (Texas Red) in bronchial, bronchiolar, and alveolar epithelial cells, IF, bar: 50 µm

**Fig. 8:**
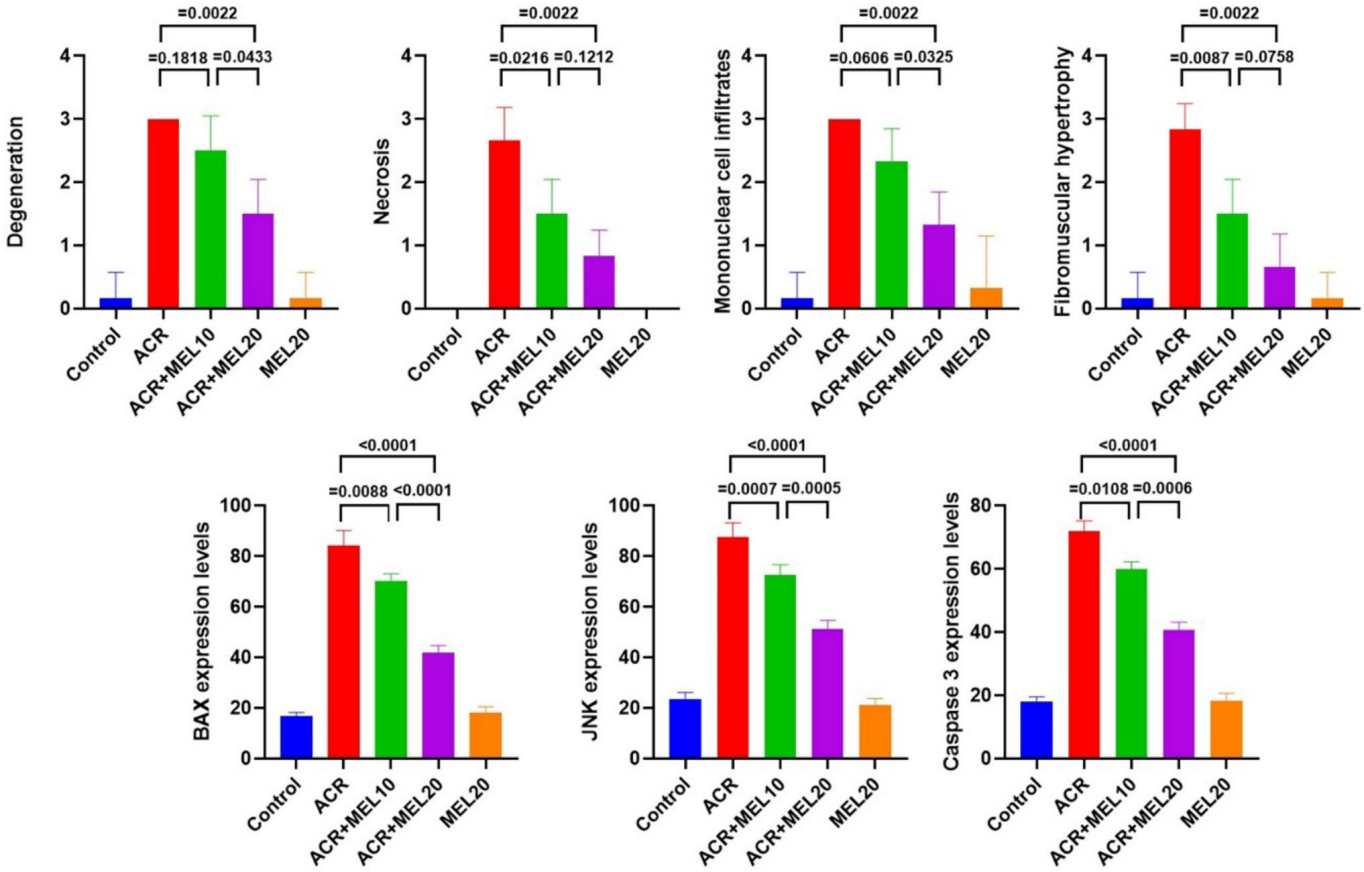
Scores of histopathological, immunohistochemical, and immunofluoresence findings in lung tissues and statistical analysis data

In the control and MEL20 groups, lung tissues were examined using immunohistochemical and immunofluoresence staining methods, and BAX, JNK, and CASP3 expressions were evaluated as negative (Figs. 6A, E and 7). In the ACR group, severe levels of intracytoplasmic BAX (Fig. 6A), JNK, and CASP3 expressions were detected in bronchial, bronchiolar, and alveolar epithelial cells (Fig. 7). In the MEL10 + ACR group, moderate levels of intracytoplasmic BAX (Fig. 6C), JNK, and CASP3 expressions were detected in bronchial, bronchiolar, and alveolar epithelial cells (Fig. 7). In the MEL20 + ACR group, mild levels of intracytoplasmic BAX (Fig. 6D), JNK, and CASP3 expressions were observed in bronchial, bronchiolar, and alveolar epithelial cells (Fig. 7). The immunohistochemical and immunofluoresence staining results and statistical analysis findings are presented in Fig. 8.

### Molecular docking results

#### Molecular docking

**Fig. 9:**
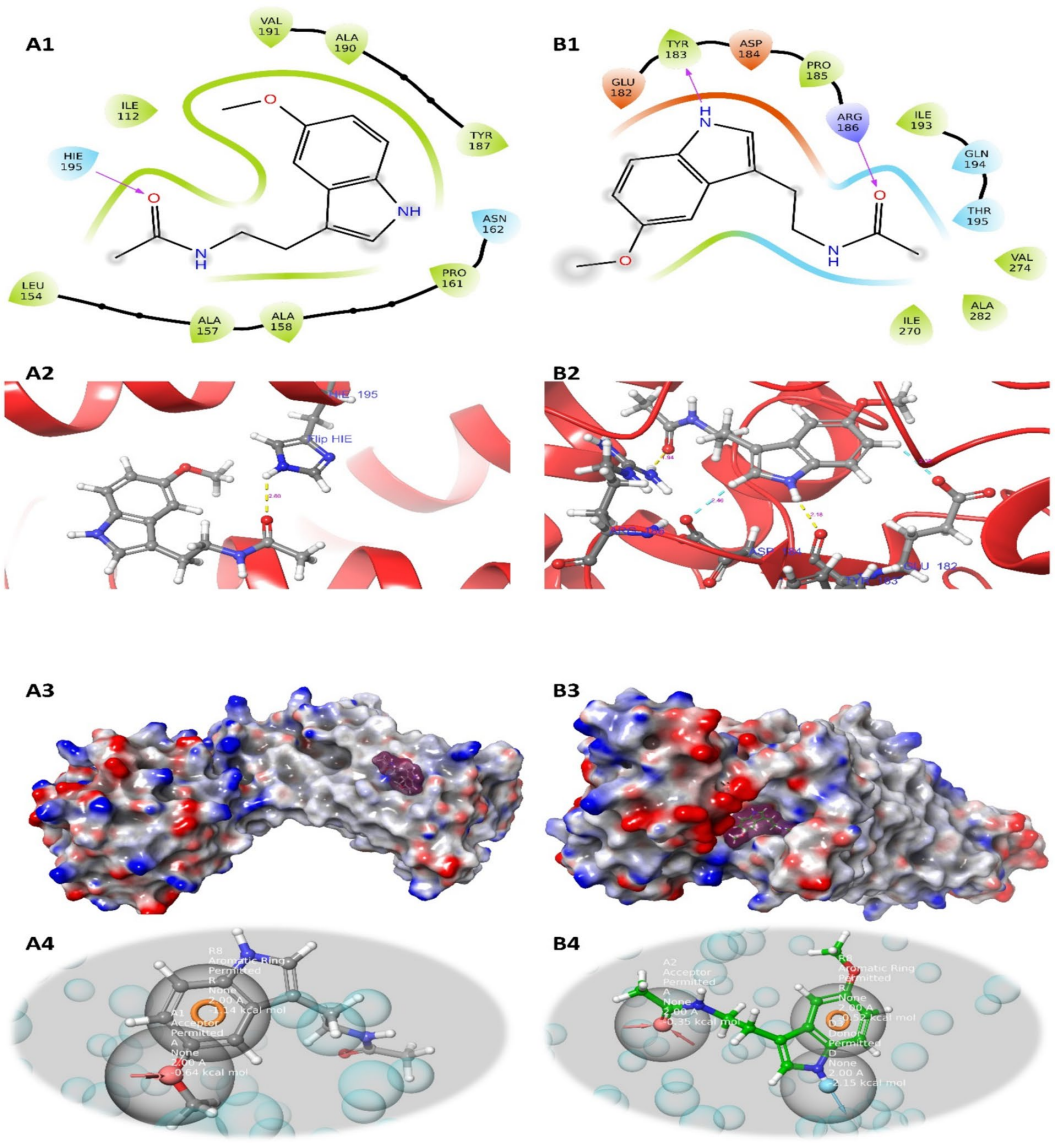
**A1** 2D image of melatonin/MT1 molecular binding, **A2** 3D image of melatonin/MT1 molecular binding, **A3** surface image of melatonin/MT1 molecular binding, **A4** 3D image of melatonin/MT1 pharmacophore mapping, **B1** 2D image of melatonin/MT2 molecular binding, **B2** 3D image of melatonin/MT2 molecular binding, **B3** surface image of melatonin/MT2 molecular binding, **B4** 3D ımage of melatonin/MT2 pharmacophore mapping

These binding scores and Glide energy values are two important parameters used to evaluate the interaction of the MEL molecule with two different target proteins (MT1 and MT2) (Fig. 9).

r_i_docking score: MEL/MT1: The interaction of MEL with the MT1 receptor is represented by a binding score of − 4.57667. MEL/MT2: The interaction of MEL with the MT2 receptor is evaluated with a binding score of − 4.80849. Lower (negative) scores may indicate stronger and preferred interactions. This suggests that MEL exhibits a positive interaction with both MT1 and MT2 receptors (Table 2).

**Table 2:** Molecular binding analysis scores

Binding scores	Melatonin/MT1	Melatonin/MT2
r_i_docking_score: The score representing the interaction of the molecular compound with the target protein	− 4.57667	− 4.80849
r_i_glide_energy: Total energy resulting from the Glide simulation. It represents molecular interaction and stability	− 26.1183	− 26.8533

r_i_glide energy: MEL/MT1: The total energy value of MEL’s interaction with the MT1 receptor is − 26.1183. MEL/MT2: The total energy value of MEL’s interaction with the MT2 receptor is − 26.8533. These values are a measure of molecular interactions and stability during the Glide simulation. A lower energy value can indicate a stable and strong interaction. These values suggest that the interaction with both MT1 and MT2 receptors is similarly stable and strong (Table 2).

These binding complexes and amino acid interactions show the binding details of the MEL molecule with the MT1 and MT2 receptors.

##### Melatonin/MT1

MEL formed a hydrogen bond with the MT1 receptor. This interaction occurred with the HIE 195 amino acid, and the bond was formed between H:7818 atom and O:7821 atom with a distance of 2.60 Å. Hydrogen bonds indicate a strong and specific interaction between molecules (Table 3).

**Table 3:** Detailed table showing the distance between the amino acid to which the ligand binds and the atoms it interacts with, as determined by molecular docking analysis

Docking complex	Aminoacid	Atom1 (receptor)	Atom2 (ligand)	Distance (angstrom = Å)
Melatonin/MT1	Hydrogen bonds with HIE 195	H:7818	O:7821	2.60 Å
Melatonin/MT2	Hydrogen bonds with ASP 184	O:1737	H:7125	2.46 Å
	Aromatic H bond with GLU 182	O:6165	H:7131	2.39 Å
	Hydrogen bonds with ARG 186	H:4714	O:7107	1.94 Å
	Aromatic H bond with TRY 183	O:1722	H:7128	2.18 Å

##### Melatonin/MT2

Various interactions were observed between MEL and the MT2 receptor: MEL formed a hydrogen bond with the ASP 184 amino acid. In this interaction, the distance between the O:1737 atom and H:7125 atom is 2.46 Å. An aromatic hydrogen bond was formed with GLU 182. The distance between the O:6165 atom and H:7131 atom is 2.39 Å. A hydrogen bond was formed with ARG 186. The distance between the H:4714 atom and O:7107 atom is 1.94 Å. An aromatic hydrogen bond was formed with TRY 183. The distance between the O:1722 atom and H:7128 atom is 2.18 Å (Table 3).

This dataset contains the results of a pharmacophore mapping analysis conducted to evaluate the interactions of the MEL molecule with the MT1 and MT2 receptors. These results summarize the data obtained from the pharmacophore mapping analysis and are used to better understand the interactions of the MEL molecule with the MT1 and MT2 receptors (Table 4).

**Table 4:** Pharmacophore mapping analysis results (A = acceptor, D = donor, H = hydrophobic, N = negative ionic, P = positive ionic, R = aromatic rings)

Features	Melatonin/MT1		Melatonin/MT2		
Rank	1	2	1	2	3
Feature_label	N3	R4	D3	R8	A2
Score	− 0.77	− 0.53	− 2.15	− 0.52	− 0.35
*X*	−33.7645	− 29.7185	37.2636	34.9406	30.2242
*Y*	24.9889	27.7503	− 44.3037	− 46.2614	−42.1675
*Z*	5.6345	2.4011	131.6744	133.431	129.5915
Type	N	R	D	R	A
Num	2	3	2	7	7
From_chemscore	0	0	0	0	0
Source	HBond	RingChemscoreHphobe	HBond + PhobEnHB	RingChemscoreHphobe	HBond

#### Molecular dynamic

Here, various analysis graphs are presented to evaluate the interactions of the MEL molecule with MT1 and MT2 receptors.

##### Root mean square deviation (RMSD) graph

**Fig. 10:**
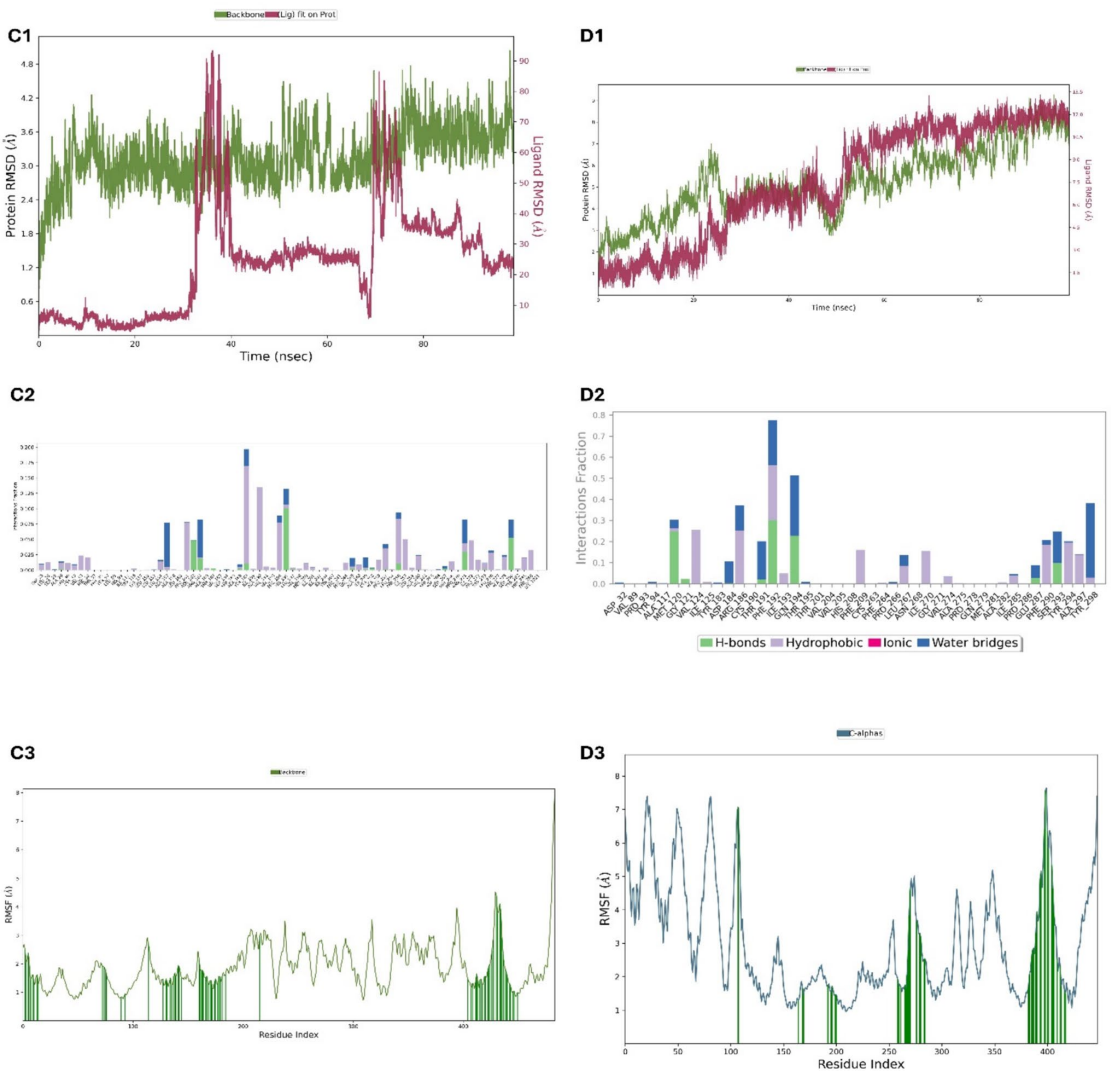
**C1** Melatonin/MT1 root mean square deviation (RMSD) graph, **C2** melatonin/MT1 protein–ligand ınteractions (hydrogen bonds, hydrophobic, ıonic, and water bridges) graph, **C3** melatonin/MT1 root mean square fluctuation (RMSF) graph, **D1** melatonin/MT2 root mean square deviation (RMSD) graph, **D2** melatonin/MT2 protein–ligand ınteractions (hydrogen bonds, hydrophobic, ıonic, and water bridges) graph, **D3** melatonin/MT2 root mean square fluctuation (RMSF) graph

RMSD shows the binding process of the ligand (MEL) to the receptor protein backbone over time. The RMSD graph for MT1 and MT2 receptors represents the ligand’s binding process to the receptor protein backbone (Fig. 10).

##### Protein–ligand ınteractions (hydrogen bonds, hydrophobic, ıonic, and water bridges) graph

This graph displays the various types of interactions (hydrogen bonds, hydrophobic ınteractions, ıonic ınteractions, and water bridges) between MEL and the MT1 and MT2 receptors over time (Fig. 10).

##### Root mean square fluctuation (RMSF) graph

RMSF indicates which regions of the protein or Cα structure are in contact with the ligand and when this contact occurs. These graphs provide a detailed representation of the interaction between MEL and the MT1 and MT2 receptors. High RMSF values may suggest that certain regions are more flexible or variable (Fig. 10).

## Discussion

ACR is a toxic molecule that is distributed throughout the body via the bloodstream due to its presence in certain foods and its high solubility in water (Besaratinia & Pfeifer 2007). It has been reported that ACR causes genotoxicity (Altinoz & Turkoz 2014), neurotoxicity (Goudarzi et al. 2019), testicular toxicity (Shahrzad et al. 2020), hepatotoxicity, and nephrotoxicity (Kandemir et al. 2020) in laboratory animals. Additionally, ACR has been shown to induce apoptosis, inflammation, and DNA damage in lung tissue and cause alterations in alveolar epithelial tissue (Demir et al. 2022). Oral exposure to ACR has been demonstrated to result in inflammation in the lungs, hyperplasia in alveolar epithelia, and hypertrophy in bronchiolar epithelial cells (Batoryna et al. 2019). Recently, it has been reported that oral administration of ACR to mice alters the lung microstructure, exacerbates bronchial inflammation and asthma, and may impair lung function (Batoryna et al. 2019; Hajimohammadi et al. 2020). For these reasons, in our study, we investigated the potential effects of MEL on oxidative stress, inflammation, and apoptosis in the lungs caused by ACR.

During the physiological activities of cells, chemical compounds called oxidants are produced. The level of oxidants is controlled by antioxidants, and if the balance between these two is disrupted, oxidative stress occurs. Oxidative stress is known to play a role in the pathogenesis of many diseases (Kara et al. 2016). ACR leads to the formation of reactive oxygen species (ROS), which cause the breakdown of polyunsaturated fatty acids in cell membranes, lipid membrane peroxidation, and the formation of malondialdehyde (MDA) as an end product (Sengul et al. 2021). It has been reported that ACR induces lipid peroxidation in erythrocytes, increasing MDA levels (Catalgol et al. 2009). MEL is a small-sized and amphiphilic molecule. Due to these properties, it can easily penetrate cells and exert antioxidant effects in both lipid and aqueous environments. Through this effect, MEL can reduce cellular oxidative stress directly or indirectly (Sheikholeslami et al. 2021). ACR causes oxidative stress, which increases ROS in tissues while reducing antioxidant enzyme levels in cells (Alturfan et al. 2012). Antioxidants combat the harmful effects of ROS. These antioxidants include SOD, CAT, GSH, and GPx (Asaduzzaman Khan et al. 2010). As a free radical scavenger, MEL directly eliminates ROS and reactive nitrogen species (RNS) while stimulating antioxidant enzymes, thereby enhancing its antioxidant efficiency. Studies have reported that MEL provides protective effects against ACR-induced oxidative damage in rats (Tozan-Beceren et al. 2012). In light of all this information, our findings demonstrate that MEL mitigates the oxidative stress effects caused by ACR in lung tissue by preventing the increase in MDA levels and the decrease in GSH, SOD, GPx, and CAT levels.

The Nrf2/HO-1 signaling pathway protects cells against inflammation and oxidative stress by regulating the expression of different antioxidant genes in response to various stimuli (Sahin et al. 2010). Nrf2 functions as a critical transcription factor to counteract oxidative stress by promoting the transcription of the HO-1 enzyme. The activation of Nrf2 is suppressed by the Kelch-like ECH-associated protein (Keap1). When tissue damage occurs, the Keap1-Nrf2 interaction becomes unstable, leading to Nrf2 activation, nuclear translocation, and subsequent regulation of genes, including HO-1 (Nath & Agarwal 2020). The Nrf2 pathway plays an important role in regulating the expression of detoxifying and antioxidant genes essential for cell survival. Recent studies have shown that Nrf2 activation plays a significant protective role in acute lung injury (Reddy et al. 2009; Zhou et al. 2015). HO-1 has an antioxidant role and is one of the key cytoprotective mechanisms activated under conditions that induce cellular stress (Sahin et al. 2010). Under normal conditions, HO-1 expression is low in cells. However, in situations causing oxidative stress, HO-1 expression accelerates, providing cellular protection. HO-1, which plays an important role in protecting macrophages, is strongly expressed in lung macrophages, acting as a critical defense mechanism against lung inflammation and oxidative damage (Sohrabi et al. 2021). Based on all this information, a study examining the effects of ACR on lung damage reported that NRF2 and HO-1 mRNA expression levels were lower in ACR-exposed groups compared to other groups (Yesildag et al. 2022). In a study conducted by Zhou et al., MEL treatment inhibited the activation of JNK, p38, and NF-κB while enhancing Nrf2 activation. This suggests that the protective role of MEL in hepatic I/R-induced lung injury occurs partly through the inhibition of JNK, p38 MAPK, and NF-κB and the upregulation of Nrf2 signaling pathways (Zhou et al. 2015). In our study, ACR reduced Nrf2 and HO-1 levels. However, in groups treated with MEL, increased levels of Nrf2 and HO-1 were observed. This indicates that the Nrf2/HO-1 signaling pathway may contribute to the protective role of MEL in ACR-induced lung damage.

Excessive ROS production in cells triggers the release of inflammatory signaling molecules. The activation of inflammation in oxidative stress–affected tissues occurs through various transcription factors (Kany et al. 2019). NF-κB is a transcription factor involved in immune and pro-inflammatory responses and is activated by oxidative stress. NF-κB plays a key role in the gene regulation and activation of pro-inflammatory cytokines such as TNF-α, IL-1β, IL-6, COX-2, and iNOS, making it a highly significant therapeutic signaling pathway (Caglayan et al. 2018; Sengul et al. 2021). The anti-inflammatory effects of MEL occur by blocking the NF-κB signaling pathway (Zhang et al. 2016). MEL functions to reduce oxidative stress, inactivate NF-κB, and downregulate inflammatory cytokines. By inhibiting COX-2 and NF-κB, MEL can reduce the release of inflammatory cytokines and chemokines (Sheikholeslami et al. 2021). NF-κB plays a crucial role in regulating COX-2 expression. ACR treatment increases NF-κB expression, leading to COX-2 induction. In a study conducted by Sengul et al., ACR was shown to activate NF-κB, increasing the production of pro-inflammatory cytokines and thereby triggering inflammation (Sengul et al. 2021). Cytokines are secretory proteins smaller than 40 kDa, produced by nearly all cells to regulate and mediate immune responses. The release of pro-inflammatory cytokines activates immune cells, leading to the production and release of additional cytokines. IL-1β, released from hematopoietic cells, and TNF-α, released from immune cells, are key inflammatory factors. IL-6 is often used as a marker because of its central role in triggering and sustaining the inflammatory response (Kany et al. 2019). IL-10 is an anti-inflammatory cytokine that inhibits NF-κB activation and plays an essential role in preventing inflammation (Wang et al. 1995). MEL also enhances anti-inflammatory cytokine levels. Raghavendra et al. reported that MEL increases IL-10 levels while reducing TNF-α levels (Raghavendra et al. 2001). Studies have shown that MEL exerts an anti-inflammatory effect by preventing an increase in pro-inflammatory cytokine levels and a decrease in anti-inflammatory cytokine levels (Ibaokurgil et al. 2023). In our current study, NF-κB was activated in response to oxidative stress in ACR-exposed rats, leading to the production of pro-inflammatory cytokines such as TNF-α, IL-1β, IL-6, and COX-2, resulting in lung damage. MEL was observed to inhibit ACR-induced NF-κB, TNF-α, IL-1β, IL-6, and COX-2 activation. In ACR-treated rats, IL-10 activity was suppressed, contributing to tissue damage. However, MEL dose-dependently increased IL-10 activity, preventing tissue damage.

One of the key factors driving inflammation is the MAPK family. Comprising ERK, JNK, and p38 signaling pathways, MAPK is known to activate NF-κB by disrupting the IκB-NF-κB complex. The MAPK and NF-κB pathways play crucial roles in the regulation of apoptosis and the expression of various inflammatory protein genes. Due to their roles in inflammation, therapeutic agents targeting the MAPK and NF-κB pathways offer significant advantages in treatment. Essential molecules responsible for inflammatory responses and cell survival include JNK, p38-MAPK, and the transcription factors NF-κB and Nrf2. Once activated, NF-κB and Nrf2 are translocated to the nucleus, where they enhance the gene expression of pro-inflammatory mediators and detoxifying and antioxidant enzymes, such as HO-1 (Gur et al. 2021; Zhou et al. 2015). It is well-known that p38 MAPK is activated and phosphorylated by oxidative stress. Tissue damage and other external stimuli will trigger the release of multiple pro-inflammatory cytokines, such as TNF-α, IL-1β, and IL-6, followed by the activation of p38 (Chaparro-Huerta et al. 2008). The JNK pathway, which is activated in response to stress, plays a significant role in apoptosis and inflammation (Ibaokurgil et al. 2023). In a study conducted by Gur et al., increased p38 MAPK levels were observed in ACR-induced toxicity (Gur et al. 2021). Additionally, in another study, MEL inhibited JNK, p38, and NF-κB activation, while Nrf2 activation was further enhanced with MEL treatment (Zhou et al. 2015). In a separate study, ACR was shown to increase JNK expression in rat kidney tissues, whereas MEL treatment dose-dependently suppressed JNK expression (Ibaokurgil et al. 2023). In our study, ACR increased p38 MAPK activity and JNK expression in rat lung tissues, whereas MEL treatment dose-dependently suppressed p38 MAPK activity and JNK expression. Throughout our study, findings indicate that the MAPK, NF-κB, and Nrf2 signaling pathways may contribute to the therapeutic role of MEL in ACR-induced lung injury.

Neutrophils and macrophages further exacerbate oxidative stress and inflammation in the lungs. Pro-inflammatory cytokines increase toxicity in the lungs and other target organs through iNOS. In a study on acute endotoxin–induced lung injury, treatment with iNOS inhibitors was reported to suppress the production of pro-inflammatory cytokines. Treatments using antioxidants and iNOS inhibitors have been shown to alleviate lung toxicity caused by different agents. MEL, a multifunctional antioxidant, directly neutralizes a variety of free radicals and reactive oxygen and nitrogen species. By reducing free radical production, MEL crosses biological membranes and reaches the cell nucleus, thereby enhancing its protective effect against oxidative stress. Additionally, MEL acts as an iNOS inhibitor. Topal et al. (2005) reported that MEL blocked iNOS activation in cyclophosphamide-induced bladder toxicity. Consequently, MEL effectively inhibits nitric oxide (NO) production (Macit et al. 2013; Topal et al. 2005). While NO helps repair damaged tissue by triggering inflammation, it can also increase oxidative stress and cause cellular damage. Furthermore, ACR can increase NO production by enhancing the activity of enzymes such as iNOS. Inhibition of COX-2 and iNOS is considered an effective approach for protecting against inflammatory diseases (Caglayan et al. 2018). In light of this information, our study demonstrated that ACR increased iNOS activity in rat lung tissues, whereas MEL treatment dose-dependently suppressed iNOS activity.

MPO is an enzyme that plays a significant role in the production of ROS associated with inflammation and contributes to the synthesis of NO, the complement system, and pro-inflammatory factors. Certain compounds, such as ACR, can increase the activity of enzymes like MPO. Previous studies have shown increased MPO activity in the liver of mice exposed to ACR (Er et al. 2020). Our study supports and expands upon these findings. We observed a significant increase in MPO levels in the lungs of rats exposed to ACR. However, as MEL doses increased, this elevation was notably reduced. These results highlight the potential of MEL as a protective agent against ACR-induced oxidative stress and inflammation.

Programmed cell death, influenced by internal and external factors, is called apoptosis. CASP3 is a protease that plays an essential role in the early stages of apoptosis (Sengul et al. 2021). One of the most critical factors in the execution of the apoptotic process is the activation of caspase-3. It is known that caspase-3 is activated as a result of an increase in cytochrome-c, Bax, and p38 MAPK, along with a decrease in Bcl-2 expression (Gur et al. 2021). A toxicity study demonstrated that ACR significantly increased cytoplasmic caspase-3 expression, while MEL dose-dependently prevented this increase (Ibaokurgil et al. 2023). Additionally, MEL prevents lung damage caused by hepatic ischemia–reperfusion through its anti-inflammatory and anti-apoptotic effects (Zhou et al. 2015). ACR, on the other hand, has been shown to activate caspase-3 (Sengul et al. 2021). In our study, the caspase-3 parameter was analyzed both biochemically and immunofluorescently. The findings revealed that ACR increased caspase-3 expression, while MEL dose-dependently reduced caspase-3 expression.

Bax is a member of the Bcl-2 family and triggers cell apoptosis. The activation of Bax leads to its translocation to the mitochondria, disrupting the membrane’s permeability. This change triggers the release of cytochrome c and the activation of caspases, initiating the apoptotic process. Studies have shown that ACR increases Bax levels, triggers the release of cytochrome c, and activates caspase-3 as a result (Gur et al. 2021). In a study by Emin and colleagues, it was also observed that the substance they used reduced the high Bax levels caused by ACR through its anti-apoptotic effects (Sengul et al. 2021). Our findings are consistent with these studies, showing that MEL reduces Bax levels, inhibits caspase-3 activation, and provides protection against the apoptosis and oxidative damage induced by ACR. Based on all this information, we can state that MEL inhibits the Bax/Bcl-2/caspase-3 pathway by reducing cytochrome c release, thus exhibiting anti-apoptotic properties.

It has been shown that ACR causes severe degenerative and necrotic damage in lung tissue, and this damage has been confirmed through histopathological examinations. In contrast, it has been observed that MEL significantly reduces the damage caused by various toxic agents in the lungs (Macit et al. 2013; Yesildag et al. 2022). Our histological analyses revealed significant differences among the experimental groups. The control and MEL20 groups exhibited normal lung structures without any adverse effects, while the ACR group showed severe degeneration, necrosis, mononuclear cell infiltration, and thickening of the interalveolar septa. In the MEL10 + ACR and MEL20 + ACR groups, similar changes to the ACR group were observed, but these changes were less severe. Immunohistochemical and immunofluorescent analyses confirmed these findings, with high expression of intracytoplasmic Bax, caspase-3, and JNK in the ACR group, while these markers were not expressed in the control and MEL20 groups. These results highlight the protective role of MEL in reducing ACR-induced lung damage and demonstrate its potential to alleviate oxidative stress and inflammation in the lungs. Therefore, histopathological evidence strongly supports the efficacy of MEL against ACR-induced pulmonary damage.

MEL is known to be an effective hormone that influences many cellular mechanisms regulating biological processes such as oxidative stress, inflammation, and apoptosis. We evaluated the in silico analyses of MEL’s interaction with MT1 and MT2 receptors. MEL can contribute to the reduction of oxidative stress by regulating levels of MDA, GSH, Nrf2, and HO-1. These regulatory processes can occur through MT1 and MT2 receptors (Han et al. 2019). MEL can enhance the activity of antioxidant enzymes such as SOD, GPx-1, and CAT through a mechanism mediated by MT1 and MT2 receptors, thereby reducing the effects of oxidative stress (Fang et al. 2019). MEL can exhibit anti-inflammatory effects by regulating inflammatory biomarkers such as TNF-α, IL-1β, NF-κB, IL-10, iNOS, IL-6, and COX-2. This process can also occur through MT1 and MT2 receptors (Ge et al. 2019). Apoptotic markers such as p38 MAPK and caspase-3 may interact with MEL to contribute to the regulation of cellular apoptosis. These interactions generally occur through MT1 and MT2 receptors (Boiko et al. 2022). Consequently, MEL, through its interaction with MT1 and MT2 receptors, can regulate cellular processes like oxidative stress, antioxidant enzyme activity, inflammation, and apoptosis, thereby offering protective effects on health. These interactions can contribute to maintaining cellular balance, preventing or treating various pathological conditions, and may show beneficial effects in reducing ACR-induced lung damage.

In conclusion, alveolar cells, despite their strong antioxidant defenses, are prone to oxidative damage due to their constant exposure to oxygen and various oxidants. The fragile structure of oxidative balance in the lungs can be disrupted by inhaled oxidants, harmful substances in circulation, and locally produced free radicals. ACR is a potential carcinogen that increases this susceptibility by triggering oxidative stress and inflammation. As a result, it can lead to severe damage in lung tissue, respiratory diseases, and an increased risk of lung cancer. On the other hand, our study demonstrates that ACR causes oxidative stress, inflammation, apoptosis, and tissue damage in the lungs of rats. MEL treatment has shown a protective role against ACR-induced lung damage due to its antioxidant, anti-inflammatory, and anti-apoptotic effects. However, further detailed and comprehensive studies are needed to investigate the therapeutic effects of MEL on damage caused by ACR in other organs.

## Data Availability

All source data for this work (or generated in this study) are available upon reasonable request.
